# Reduced migration of MLH1 deficient colon cancer cells depends on SPTAN1

**DOI:** 10.1186/1476-4598-13-11

**Published:** 2014-01-24

**Authors:** Inga Hinrichsen, Benjamin Philipp Ernst, Franziska Nuber, Sandra Passmann, Dieter Schäfer, Verena Steinke, Nicolaus Friedrichs, Guido Plotz, Stefan Zeuzem, Angela Brieger

**Affiliations:** 1 Medical Clinic I, Biomedical Research Laboratory, Goethe-University, Frankfurt a.M, Germany; 2 ENT Department, Goethe-University, Frankfurt a.M, Germany; 3 Institute of Human Genetics, Goethe-University, Frankfurt a.M, Germany; 4 Institute of Human Genetics, University of Bonn, Bonn, Germany; 5 Institute of Pathology, University of Cologne, Cologne, Germany

**Keywords:** DNA mismatch repair, MLH1, SPTAN1, Cytoskeletal proteins, Cellular mobility

## Abstract

**Introduction:**

Defects in the DNA mismatch repair (MMR) protein MLH1 are frequently observed in sporadic and hereditary colorectal cancers (CRC). Affected tumors generate much less metastatic potential than the MLH1 proficient forms. Although MLH1 has been shown to be not only involved in postreplicative MMR but also in several MMR independent processes like cytoskeletal organization, the connection between MLH1 and metastasis remains unclear. We recently identified non-erythroid spectrin αII (SPTAN1), a scaffolding protein involved in cell adhesion and motility, to interact with MLH1. In the current study, the interaction of MLH1 and SPTAN1 and its potential consequences for CRC metastasis was evaluated.

**Methods:**

Nine cancer cell lines as well as fresh and paraffin embedded colon cancer tissue from 12 patients were used in gene expression studies of SPTAN1 and MLH1. Co-expression of SPTAN1 and MLH1 was analyzed by siRNA knock down of MLH1 in HeLa, HEK293, MLH1 positive HCT116, SW480 and LoVo cells. Effects on cellular motility were determined in MLH1 deficient HCT116 and MLH1 deficient HEK293T compared to their MLH1 proficient sister cells, respectively.

**Results:**

MLH1 deficiency is clearly associated with SPTAN1 reduction. Moreover, siRNA knock down of MLH1 decreased the mRNA level of SPTAN1 in HeLa, HEK293 as well as in MLH1 positive HCT116 cells, which indicates a co-expression of SPTAN1 by MLH1. In addition, cellular motility of MLH1 deficient HCT116 and MLH1 deficient HEK293T cells was impaired compared to the MLH1 proficient sister clones. Consequently, overexpression of SPTAN1 increased migration of MLH1 deficient cells while knock down of SPTAN1 decreased cellular mobility of MLH1 proficient cells, indicating SPTAN1-dependent migration ability.

**Conclusions:**

These data suggest that SPTAN1 levels decreased in concordance with MLH1 reduction and impaired cellular mobility in MLH1 deficient colon cancer cells. Therefore, aggressiveness of MLH1-positive CRC might be related to SPTAN1.

## Background

The most important DNA mismatch repair (MMR) protein commonly dysregulated in colon cancer is MLH1. MLH1 is the main component of the heterodimer MutLα, formed by MLH1 and PMS2. Germline mutations in MLH1 are responsible for 50% of a hereditary form of colorectal cancer (CRC) called Lynch syndrome [1]. In addition, 13-15% of sporadic CRCs are caused by MLH1 deficiency based on somatic promotor hypermethylation [2,3].

Looking at functionality, MutLα is mainly involved in the correction of base-base mismatches and insertion-deletion loops resulting from defective DNA replication [4]. Besides, recent studies suggest that MLH1 also participates in other important fundamental cellular functions beyond its primary role in MMR, e.g., the regulation of cell cycle checkpoints and apoptosis [5], but also in meiotic reciprocal recombination and meiotic mismatch repair [6]. Several MLH1 interacting proteins have been published, which might be essential for signaling DNA damages to different cellular processes [7-11]. Amongst them we identified non-erythroid spectrin αII (SPTAN1) as a novel interaction partner of MLH1 and found evidence for the involvement of both proteins in cytoskeletal and filamental organization [12].

SPTAN1 belongs to a superfamily of F-actin cross-linking proteins (scaffolding proteins) which, first identified as membrane-skeleton components in erythrocytes, are ubiquitously expressed in metazoan cells [13]. Spectrins contribute to cell adhesion and migration [14], interact with structural and regulatory proteins [15-17] and are involved in the regulation of DNA repair [18,19]. Deregulation of spectrins, especially of SPTAN1 seriously affects cellular behavior and promotes tumor progression. Upregulated SPTAN1, e.g., was demonstrated in various types of tumors [20-23] and shown to be associated with local aggressiveness and metastic behavior of soft tissue carcinomas [24]. Moreover, enhanced SPTAN1 was linked to tumor progression and malignancy in ovarian cancer [25] and described to be involved in the carcinogenesis of sporadic CRC [21].

After i) identification of MLH1-SPTAN1 protein-protein interaction [12], ii) knowledge of MLH1 capacity to stabilize its partner proteins [26,27] and iii) indications that MLH1 deficient tumors are less aggressive and distant metastasis are less common than in MMR proficient forms [28,29], we propose a MLH1 dependent role of SPTAN1 for cellular motility, metastasis and aggressiveness of CRC.

Using different MLH1 deficient and proficient cell lines, paraffin embedded as well as fresh tumor tissue, we show for the first time that MLH1 deficiency decreases SPTAN1 expression with the functional consequence of impaired cellular migration.

## Results

### MLH1 influences SPTAN1 expression and cellular localization

It has been shown that the presence of MLH1 seems to be not only most important for PMS2 stabilization [30] but also for other interacting partner proteins [26]. Since we previously identified protein-protein interaction of MLH1 and SPTAN1 [12] and verified this interaction using HEK293 cells (Additional file 1), we analyzed the influence of MLH1 on the protein level of interacting SPTAN1 by using nine different human cancer cell lines: six MLH1 proficient and three MLH1 deficient ones. The MLH1 proficient cell lines were the human epitheloid cervix carcinoma cell line HeLa, the human embryonic kidney cell line HEK293, two stably MLH1 transfected clones of the colorectal carcinoma cell line HCT116, HCT116 mlh1-2 and HCT116 mlh1-3 [31] as well as the colorectal carcinoma cell lines SW480 and LoVo. We compared the SPTAN1 expression level of these cell lines with their corresponding MLH1 deficient cell lines, HEK293T [32] or the stably pcDNA3.1+ mock control transfected HCT116 mlh0-1 cell line [31], respectively and with the MLH1 deficient colorectal cancer cell line RKO using Western blot analysis.

**Figure 1:**
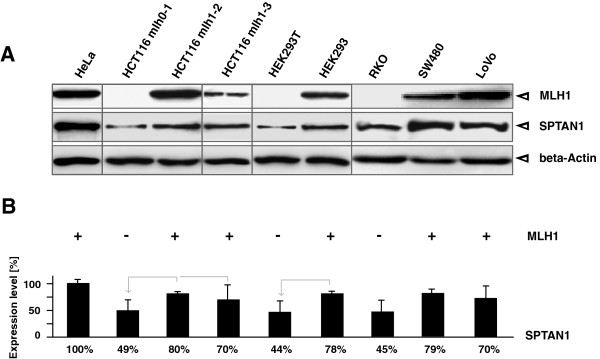
**MLH1 deficient cell lines show reduced SPTAN1 expression.** To determine a potential influence of MLH1 on SPTAN1 expression different MLH1 proficient and MLH1 deficient cell lines were analyzed by **(A)** Western blotting using anti-MLH1 or anti-SPTAN1, respectively, controlled by beta-Actin detection. **(B)** Amounts of SPTAN1 were assessed by measuring the signal intensities of protein bands with Multi Gauge V3.2 software. Graphs indicate the results (mean ± S.D.) of at least four independent experiments. Comparability was facilitated by setting SPTAN1 levels of HeLa (lane 1) as 100%. Shared identity of MLH1 proficient with MLH1 deficient sister cell lines is assigned by grey arrows.

As shown in Figure 1, MLH1 deficient cell lines (Figure 1, lanes 2, 5 and 7) showed significantly reduced SPTAN1 expression in comparison to the MLH1 proficient lines (Figure 1, lanes 3, 6, 8 and 9).

Most prominent SPTAN1 expression level was detectable in MLH1 proficient HeLa cells (Figure 1A, lane 1) which was used as the benchmark for the quantification of SPTAN1 expression of all tested cell lines and set to 100% (Figure 1B, lane 1).

SPTAN1 expression of HCT116 mlh0-1 (Figure 1, lane 2) was only 49% compared to HeLa and 31% less than the SPTAN1 level of the HCT116 mlh1-2 clone (Figure 1, lane 3). Even weakly MLH1 expressing HCT116 mlh1-3 cells (Figure 1, lane 4) showed 21% stronger expression of SPTAN1 than the MLH1 deficient mock control (Figure 1, lane 2).

In MLH1 deficient HEK293T cells (Figure 1, lane 5) SPTAN1 expression was only 44% in comparison to HeLa and 34% lower than the MLH1 proficient HEK293 (Figure 1, lane 6). Moreover, MLH1 deficient RKO cells (Figure 1, lane 7) expressed only 44% of SPTAN1 in comparison to HeLa and significant less than the MLH1 proficient SW480 and LoVo cells (Figure 1, lane 8 and 9).

### siRNA knock down of MLH1 leads to reduction of SPTAN1 mRNA

**Figure 2:**
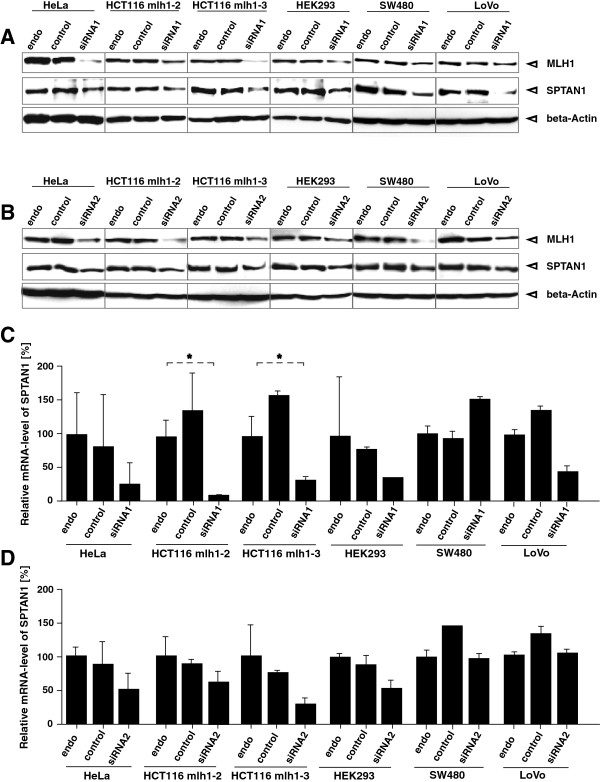
**Influence of MLH1 knock down by siRNA treatment.** Direct connection of MLH1 on SPTAN1 was studied in detail by comparison of untreated with transiently MLH1-specific siRNA1 **(A)** and siRNA2 **(B)** and control siRNA transfected different MLH1 proficient cell lines. Western blot analysis of MLH1 and SPTAN1 protein levels was performed after 48 h, controlled by beta-Actin detection after. In parallel, mRNA levels of MLH1 and SPTAN1 were determined by TaqMan-analysis with pairs of MLH1-, SPTAN1- or GAPDH-specific primers and MLH1-, SPTAN1- or GAPDH-specific probes, respectively, after treatment with MLH1-specific siRNA1 **(C)** and siRNA2 **(D)**. The data show that siRNA knock down of MLH1 led to impaired SPTAN1 expression and partly to reduced mRNA level of SPTAN1. Graphs indicate the results (mean ± S.D.) of at least three independent experiments.

In order to analyze the influence of MLH1 knock down on the mRNA level of SPTAN1 we determined the mRNA amount of SPTAN1 48 h after treatment in comparison to control siRNA treated cells via qRT-PCR (Figure 2C and D).

We forced MLH1 protein reduction via siRNA treatment with two different siRNAs against MLH1 using the MLH1 proficient cell lines of our panel: HeLa, HCT116 mlh1-2, HCT116 mlh1-3, HEK293, SW480 and LoVo (described above). As demonstrated by Western blot analysis (Figure 2A and B) siRNA knock down of MLH1 lead to SPTAN1 reduction after 48 h in all these cell lines (Figure 2A and B, lanes 3, 6, 9, 12, 15 and 18) whereas treatment with siRNA control had no effect on SPTAN1 expression (Figure 2A, lanes 2, 5, 8, 11, 14 and 17).

However, siRNA knock down of MLH1 led not only to the reduction of MLH1 itself but also to reduced SPTAN1 mRNA levels in four or five of the treated cell lines, respectively (Figure 2C and D, lanes 3, 6, 9, 12 and 18).

### SPTAN1 amount depends on the MLH1 level

In addition, the MLH1-SPTAN1 connection was verified by monitoring SPTAN1 expression in parallel to growing amount of MLH1. Hereby, SPTAN1 was determined in HEK293T cells transiently transfected with MLH1 from 0 h to 20 h. As shown in Additional file 2, a clear increase of SPTAN1 expression was detectable (Additional file 2A and C, lanes 6-9) slightly time-delayed to the gain of MLH1 (Additional file 2A and B, lanes 4-9).

To emphasize that MLH1 itself and not a general loss of the MMR system lead to SPTAN1 reduction, we also determined SPTAN1 levels in MMR deficient LoVo cells caused by a defect in MSH2 (the second most important Lynch syndrome associated MMR protein [33]). However, similar protein levels of SPTAN1 were detectable in MSH2 deficient as well as MSH2 overexpressing LoVo cells (Additional file 3), therefore an influence of MSH2 on SPTAN1 could be excluded.

**Figure 3:**
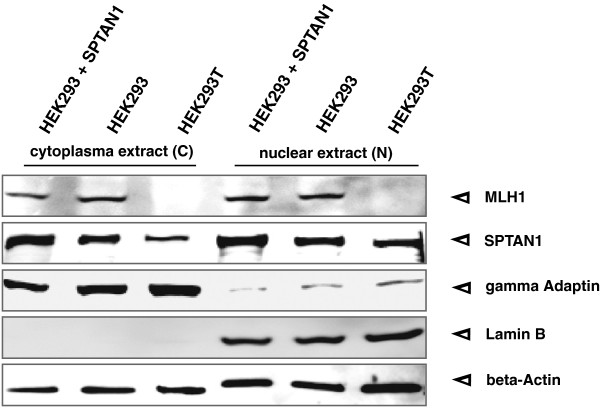
**Nuclear and cytoplasmic SPTAN1 analysis.** SPTAN1 was described to be involved in DNA repair processes. To determine cellular localization of SPTAN1, nuclear (N) and cytoplasmic (C) protein fractions of SPTAN1 overexpressing or corresponding untransfected HEK293 and HEK293T cells, cells were harvested and analyzed by Western blotting. The efficiency of the fractionation was verified by staining for Lamin B as a nuclear marker and gamma Adaptin as a cytoplasmic marker. In parallel, beta-Actin detection served for quantitative control.

Since deregulation of SPTAN1 was described to cause changed cellular SPTAN1 localization in sporadic CRCs [21], we furthermore compared the cellular distribution of SPTAN1 in nuclear and cytoplasmic fractions of MLH1 proficient SPTAN1 transfected and untransfected HEK293 as well as MLH1 deficient HEK293T cells (Figure 3). Our data show that SPTAN1 was expressed in both cell fractions of all tested cell lines (Figure 3, middle panel) and overexpression of SPTAN1 generally led to enhancement of SPTAN1 similarly in the cytoplasm as well as in the nucleus. However, while the amount of SPTAN1 was equal in the cytoplasm and the nucleus of both MLH1 proficient HEK293 variants, MLH1 deficient HEK293T showed obviously less SPTAN1 in the cytoplasm than in the nuclear fraction (Figure 3, lane 3 vs lane 6).

### MLH1 deficient colon cancer tissue showed significantly reduced SPTAN1 expression

**Figure 4:**
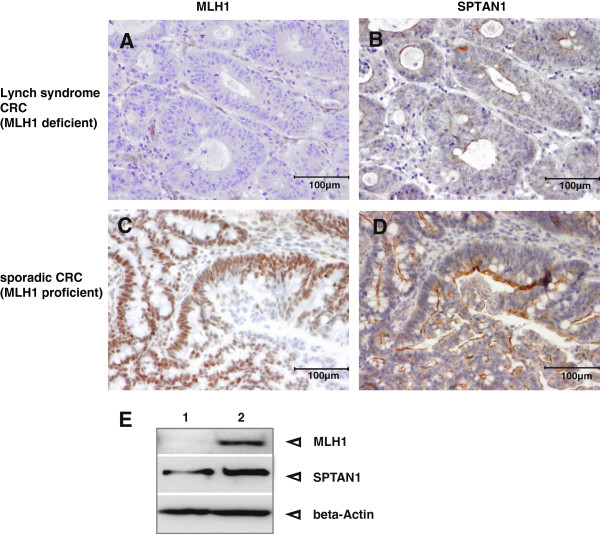
**Analysis of MLH1 deficient Lynch syndrome and MLH1 proficient sporadic CRC.** Immunhistochemistry demonstrated loss of **(A)** MLH1 as well as **(B)** significant reduction of SPTAN1 in Lynch syndrome CRC tissue whereas MLH1 and SPTAN1 was strong expressed **(C and D)** in the sporadic CRC, respectively. Magnification 100×. In addition, Western blot analysis **(E)** of MLH1 deficient fresh tumor tissue (lane 1) in comparison to normal tissue (lane 2) of MLH1 promotermethylated sporadic CRC showed a clear decrease of SPTAN1 in the MLH1 deficient tumor tissue (lane 1, middle panel). All MLH1 deficient species showed reduction of SPTAN1 expression (see Table 1).

To verify the connection of MLH1 and SPTAN1 *in vivo*, we isolated protein extract of one MLH1 deficient fresh CRC biopsy and the corresponding normal tissue from a patient. Additionally, we performed immunohistochemistry using paraffin embedded invasive growing CRC tissue from patients showing defective MLH1 expression in comparison to those with MLH1 proficiency. Hereby, we analyzed sporadic MLH1 deficient CRCs, CRCs from Lynch syndrome patients caused by MLH1 mutations and sporadic CRCs without MLH1 defect (Figure 4).

In the fresh biopsy tissue from the CRC patient, SPTAN1 was well detectable in the normal tissue (Figure 4E, lane 2) while the corresponding tumor showed reduced expression (Figure 4E, lane 1).

Immunhistochemistry data (Table 1) demonstrated a significant reduction of SPTAN1 in all MLH1 deficient tumor sections, while SPTAN1 could be clearly detected in all MLH1 proficient CRC tissues; exemplary results are shown in Figure 4B and D, respectively.

**Table 1:** Characteristics of analysed patients-tissue

**Patients**	**Gender**	**Age at diagnosis (years)**	**CRC**			**MLH1**	**SPTAN1**
			**Sporadic MLH1+**	**Sporadic MLH1-(BRAF V600E)**	**Lynch syndrome MLH1 mutation**		
Paraffin embedded tissue							
1.	m	63	x	-	-	+++	+++
2.	m	71	x	-	-	+++	+++
3.	m	46	x	-	-	++	++
4.	m	68	-	x	-	-	+
5.	m	50	-	x	-	-	+
6.	f	67	-	x	-	-	+
7.	m	49	-	x	-	-	+
8.	m	49	-	-	c.275_278delCCAG	-	+
					(p.Ala92ValfsX15)		
9.	m	57	-	-	c.1731G>A	-	+
					(p.Ser577Ser)		
10.	m	49	-	-	c.676C>T	-	+
					(p.Arg226X)		
11.	f	44	-	-	c.583A>T	-	+
					(p.Lys195X)		
Fresh tissue							
12. (N)	m	80	-	-	-	++	+++
12. (T)			-	x	-	-	+

-: deficiency; ++: moderate expression; +++: strong expression; +: weak expression; N: tumour surrounding tissue; T: tumour tissue.

### Migration assay demonstrates that MLH1 deficiency impairs cell mobility

**Figure 5:**
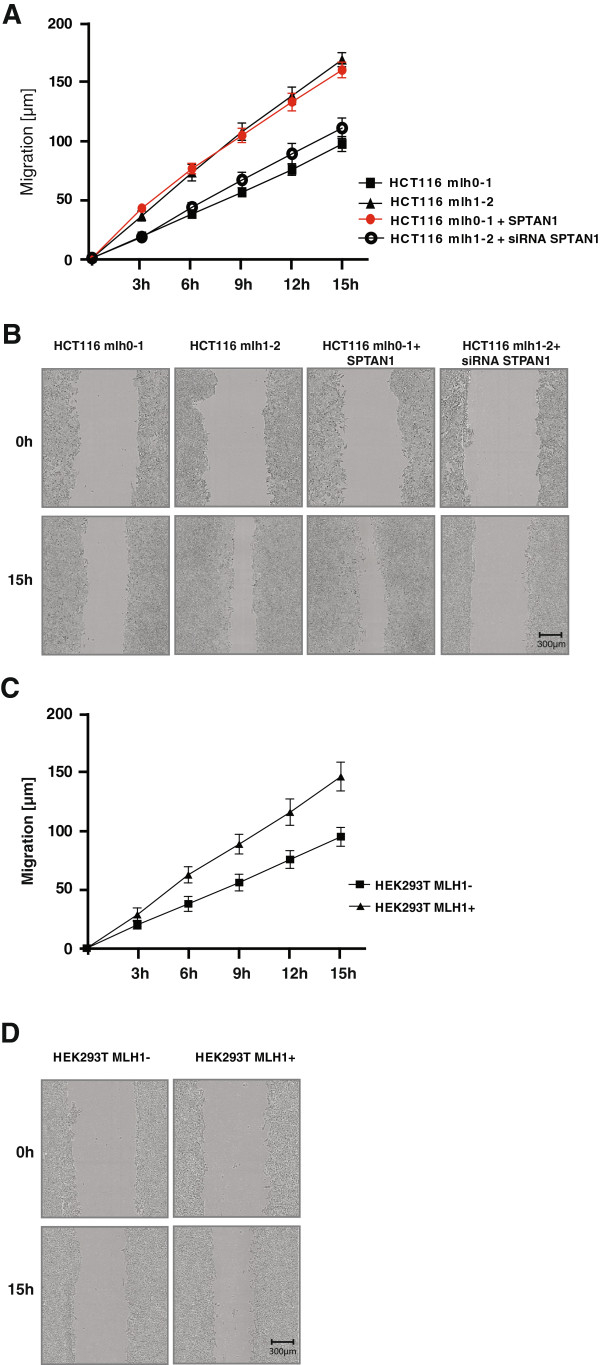
**Overexpression of SPTAN1 restores mobility of MLH1 deficient HCT116 mlh0-1 cells.** Cell migration of **(A)** HCT116 mlh0-1, HCT116 mlh1-2, SPTAN1 overexpressing HCT116 mlh0-1 and siRNA SPTAN1 treated HCT116 mlh1-2 cells or **(C)** doxycycline treated MLH1 deficient HEK293T (HEK293T MLH1-) and untreated MLH1 proficient HEK293T (HEK293T MLH1+) cells were analyzed using an IncuCyte™ microscope. Distance of migration representative of three different experiments each done in quadruplicate was analyzed at different time points (0 h, 3 h, 6 h, 9 h and 15 h). **(B and D)** Representative corresponding images were taken from each cell line immediately after scratching the cultures (0 h) and 15 h later. Original Magnification 400×. Migration of MLH1 deficient HCT116 mlh0-1 could be restored to the level of MLH1 proficient HCT116 mlh1-2 after overexpression of SPTAN1 while mobility of HCT116 mlh1-2 treated with siRNA SPTAN1 was reduced to the level of HCT116 mlh0-1. Graphs indicate the results (mean ± S.D.) of at least three independent experiments.

To test the potential functional influence of SPTAN1 reduction on cell mobility the migratory rate of MLH1 deficient and MLH1 proficient cells was compared in a migration assay by measuring the alteration of the wound width at different time points (3 h, 6 h, 12 h and 15 h). As shown in Figure 5A and B, mobility of HCT116 mlh0-1, HCT116 mlh1-2, SPTAN1 overexpressing HCT116 mlh0-1 as well as siRNA SPTAN1 treated HCT116 mlh1-2 cells grown in 96-well plates was analyzed. The mobility of MLH1 deficient HCT116 mlh0-1 cells was significantly worse than that of the MLH1 proficient HCT116 mlh1-2 sister clone (Figure 5A and B). Overexpression of SPTAN1 enhanced the migration ability of MLH1 deficient HCT116 mlh0-1 to a similar extent as detected for the MLH1 proficient sister cell line (Figure 5A and B). siRNA knock down of SPTAN1 in HCT116 mlh1-2 cells leads to a similar mobility as detected in MLH1 deficient HCT116 mlh0-1 cells (Figure 5A and B). Treatment of HCT116mlh1-2 with control siRNA showed no effect (data not shown).

This effect could also be shown by using doxycycline inducible HEK293T cells (Figure 5C and D). Doxycycline treated MLH1 deficient HEK293T cells showed worse mobility that untreated MLH1 proficient HEK293T cells (Figure 5C and D).

## Discussion

The functionality of the DNA mismatch repair protein MLH1 is apparently of great diversity. MLH1 was not only described to be involved in MMR but also in many other essential cellular processes ranging from the regulation of cell cycle checkpoints, apoptosis, meiotic reciprocal recombination, meiotic mismatch repair to cytoskeletal organization [5,6,12,34]. An active dialogue of the MMR system with the cellular cytoskeleton might be postulated since MMR deficient Lynch syndrome tumors and sporadic CRCs with MLH1 defect seem to be less aggressive than MMR proficient forms. Gryfe and coworkers e.g. tested specimens of colorectal cancer from a population-based series of 607 patients and found that lymph node metastases are less common in the MMR deficient form [28]. Moreover, Jeong et al. showed that distant metastases are less frequent in MMR deficient sporadic CRCs [29]. However, the underlying mechanisms are still unknown.

In the present study we focused on the relationship of MLH1 to cytoskeletal associated SPTAN1 [12] and found a clear correlation between MLH1 deficiency and SPTAN1 impairment in different cell lines as well as in fresh or paraffin embedded invasive growing CRC tissue. We detected SPTAN1 reduction mainly in the cytoplasm of cells which leads us to the assumption that the interaction of MLH1 and SPTAN1 might take place rather in the cytoplasm than in the nucleus [35]. However, the connection between MLH1 deficiency and SPTAN1 reduction was detectable not only on protein but partly also on mRNA level. While the MLH1 dependent decrease of SPTAN1 expression might be easily explainable by missing MLH1 protein stabilization and has been shown for other MLH1 partner proteins before [26,27], the explanation for reduced SPTAN1 mRNA levels in four of six cancer cell lines detectable after siRNA-specific down knocking of MLH1 using two different siRNAs seems to be more difficult. One might hypothesize that MLH1 and SPTAN1 are members of one complex and are functionally co-regulated. Thus, cellular monitoring of impaired MLH1 might rapidly lead to SPTAN1 reduction through an enzymatic breakdown of RNA transcripts and existing protein molecules. This assumption is supported by several publications showing that proteins of protein complexes are co-expressed both in terms of mRNA levels and expression profiles [36,37]. However, since the demonstrated siRNA results were inhomogeneous we cannot exclude unspecific off-target siRNA effects which were demonstrated to be due to the nature of RNA interference [38-40].

In addition, our data demonstrate that the MLH1 dependent reduction of SPTAN1 changed the cellular mobility and impaired the migration ability of affected cells. The observed correlation between extenuated SPTAN1 and decreased cell mobility is in line with data from Metral et al., who found a tight involvement of SPTAN1 in actin organization [41], and Sormunen and co-workers, who demonstrated that the composition and interplay of the E-cadherin/β-catenin/SPTAN1/cytoskeleton-complex is of great importance for cell-to-cell adhesion and cell shape [42]. Besides, the present observation also matches the MLH1 dependent cytoskeleton alteration of HeLa cells previously detected by us [12]. Therefore, we postulate that MLH1 dependent SPTAN1 reduction might induce changes in actin filaments and finally the cytoskeleton leading to impaired migration.

## Conclusion

In summary, our data indicate a close correlation between MLH1 deficiency, SPTAN1 reduction and impaired cell migration, which might be one feasible explanation for the reduced potential of MLH1 defective tumors to metastasize.

## Material and methods

### Patients

Paraffin-embedded tissue from 11 patients with surgically resected well characterized CRC specimens were selected for immunhistochemical analysis: 4 were from Lynch syndrome patients caused by pathogenic MLH1 mutations, 4 were sporadic CRCs with MLH1 defect caused by hypermethylation of the MLH1 promoter associated with BRAF V600E mutation and 3 tumors were MMR proficient sporadic CRCs.

Characteristics of the analyzed patients-tissue are summarized in Table 1.

MLH1 as well as SPTAN1 expression was analyzed in triplicate, respectively, of every tumor and tumor surrounding tissue using immunohistochemistry.

In parallel, a fresh (MLH1 promotor hypermethylated) tumor biopsy and the corresponding normal tissue were exemplarily taken from one CRC patient to perform Western blot analysis of MLH1 and SPTAN1.

The study was approved by the local ethic committees of Frankfurt/Main (Germany), Bonn (Germany) and Homburg Saar (Germany). All patients gave informed consent.

### Cell lines

HeLa (ATCC number CCL-2), HEK293 (ATCC number: CRL-1573) and LoVo (ATCC number: CCL-229) were purchased from American Type Culture. HEK293T cells, obtained from Dr. Kurt Ballmer (Paul Scherer Institute, Villingen, Switzerland) were grown in DMEM with 10% FCS. As previously described, MLH1 is not expressed in HEK293T [32]. Stably transfected HCT116 cells: HCT116 mlh 0-1 transfected with the pcDNA3.1+ vector and HCT116 mlh 1-2 as well as HCT116 mlh 1-3 transfected with pcDNA3.1+ containing the entire open reading frame of MLH1 obtained for Prof. Francoise Praz (Institute Gustave Roussy, Villejuif, France) were grown in DMEM with 10% FCS and hygromycin B (100 μg/ml) [31]. Doxycycline inducible HEK293T MLH1+ cells, obtained from Prof. Josef Jiricny (University of Zurich, Institute of Molecular Cancer Research, Zurich, Switzerland), were cultured in DMEM supplemented with 10% Tet System-approved fetal calf serum, hygromycin B (300 mg/ml), and Zeocin (100 mg/ml). To abrogate MLH1 expression, the cells (HEK293T MLH1-) were cultured for 4 days in the presence of doxycyclin (50 ng/ml) [43].

### Antibodies and plasmids

Anti-SPTAN1 (C-11), anti-Lamin B (C-20) and anti-MSH2 (H-300) were obtained from Santa Cruz Biotechnology (Santa Cruz, CA), anti-MLH1 (G168-728) as well as anti-gamma Adaptin (88) were from Pharmingen (BD Biosciences, United States), anti-beta Actin (Clone AC-15) was purchased from Sigma (Sigma-Aldrich, USA) and anti-SPTAN1 (MAB1622) was from Millipore (Millipore, USA).

The pcDNA3.1+/MLH1, pcDNA3.1+/PMS2 and pcDNA3.1+/MSH2 expression plasmids were described previously [27]. Full length SPTAN1 cDNA was generated from total RNA of human lymphocytes and subcloned into the eukaryotic expression vector pcDNA3.1- via the KpnI/XhoI restriction sites.

All plasmids were confirmed by sequencing and reading frames were corrected using site-directed mutagenesis, if necessary. All oligonucleotides were purchased from Sigma-Aldrich (Munich, Germany).

### siRNA treatment and real-time quantitative reverse transcription-PCR (qRT-PCR)

Different stably transfected HCT116 cells (HCT116 mlh 1-2 and HCT116 mlh 1-3), HEK293 and HeLa cells were treated with siRNA according to manufacturer’s protocol (Applied Biosystems, Foster City, CA). In brief, 100 μl of OPTI-MEM (Gibco, Biocompare, San Francisco, CA) was mixed with 5 μl siPORT NeoFx Transfection Agent (Applied Biosystems, Foster City, CA) and incubated for 10 min at room temperature. 100 μl of OPTI-MEM containing 30 nM of Silencer Validated siRNA MLH1 (siRNA1) (Applied Biosystems, ID# 119549), 5 nM of Silencer Select siRNA MLH1 (siRNA2) (Ambion, ID# 4392420), 5 nM of Silencer siRNA SPTAN1 (Applied Biosystems, ID# s13405), or 30 nM of Silencer Negative Control siRNA (Applied Biosystems, ID#1), respectively was added and incubated for further 10 min at room temperature. 1×10^5^ cells, diluted in DMEM medium with 10% FCS, were added to a final volume of 2.5 ml, the mixture was placed in a 6-well and incubated at 37°C with 5% CO_2_ for 24 h and 48 h. Thereafter, treated HCT116 mlh 1-2 and HCT116 mlh 1-3, HEK293 and HeLa cells were harvested, homogenized in Trizol reagent (Invitrogen, Carlsbad, CA) and RNA was isolated according to manufacturer’s protocol. The mRNA levels of MLH1, SPTAN1, or GAPDH (internal control) were determined by a real-time quantitative reverse transcription-PCR (qRT-PCR) assay (TaqMan) with a pair of MLH1-, SPTAN1- or GAPDH-specific primers and MLH1-, SPTAN1- or GAPDH-specific probes (#Hs00179866-m1 MLH1, #Hs00162203_m1 SPTAN1, #Hs02786624_g1 GAPDH, Applied Biosystems, Foster City, CA), respectively, on a StepOnePlus™ Real-Time PCR System (Applied Biosystems, Foster City, CA).

### Transient transfection

Transiently transfection or cotransfection of HEK293T cells was carried out as described previously [26]. In brief, HEK293T were transfected at 50-70% confluence with expression plasmids, pcDNA3.1+/MLH1, pcDNA3.1+/PMS2, pcDNA3.1-/SPTAN1 or empty pcDNA3.1+ mock control (1 μg/ml, respectively) using 10 μl/ml of the cationic polymer polyethylenimine (Polysciences, Warrington, PA; stock solution 1 mg/ml). 24 h and 48 h post transfection cells extracts were prepared for Western blot analysis.

### Nuclear and cytoplasmic protein extraction from cell culture and whole protein extraction from fresh tissue

Separation of proteins from cell cultures into nuclear and cytoplasmic fractions was carried out as previously described [35]. Briefly, cells were harvested by centrifugation (10 min, 1000 g, 4°C), cell pellets were washed twice in PBS (4°C) and diluted in 250 μl hypotonic buffer (20 mM Tris-HCl pH 7.4, 10 mM NaCl, 3 mM MgCl_2,_ 0.5 mM DTT). After incubation on ice (15 min), 5% of NP-40 (10%) was added, samples were vortexed and centrifuged (10 min, 1000 g, 4°C). Supernatants containing cytoplasmic proteins were frozen and residual pellets were resuspended in cell extraction buffer (10 mM Tris/HCl pH 7.4, 100 mM NaCl, 1 mM EDTA, 1 mM EGTA, 1 mM NaF, 20 mM Na_4_P_2_O_7_, 2 mM Na_3_VO_4_, 1% Triton X-100, 10% glycerol, 0.1% SDS, 0.5% Na-depoxycholate, 1 mM PMSF and 5% protease inhibitor cocktail (Sigma Aldrich, Munich, Germany)). Samples were incubated on ice (30 min), sonicated (10 sec) and centrifuged (13000 U/min, 4°C, 30 min). Dissolved nuclear protein fractions were frozen (-80°C).

Fresh tissue biopsy sections (à 5 mg) were removed from 80°C, washed three times with PBS (4°C), transferred into a 15 ml Dounce Homogenizer tube and diluted with 150 μl (4°C) ready to use Cell Lysis Reagent (Sigma Aldrich, Munich, Germany). Biopsies were homogenized vigorously (10 min) on ice, transferred into 1.5 ml Eppendorf tubes, sonicated (10 sec) and centrifuged (5 min, 1000 g, 4°C). Supernatants containing the proteins were placed in new Eppendorf tubes and frozen (-80°C).

### Western blot analysis

Proteins were separated on 10% polyacrylamide gels, followed by Western blotting on nitrocellulose membranes and antibody detection using standard procedures or as described previously [26].

The band intensity of SPTAN1 was quantified using Multi Gauge V3.2 program (Fujifilm, Tokyo, Japan).

All experiments were performed in quadruplicate.

### Coimmunoprecipitation

Coimmunoprecipitation was carried out as previously described [12]. For detection of endogenous protein-protein interaction coimmunoprecipitation was carried out using anti-MLH1 and anti-SPTAN1, respectively, in MLH1 proficient HEK293 cells. Immunoprecipitation with protein A/G sepharose served as negative and whole cell extract (50 μg) of HEK293 cells as positive control.

### Immunohistochemical analysis

MLH1 and SPTAN1 expression was analyzed by immunohistochemistry using paraffin embedded invasive growing MLH1 deficient or MLH1 proficient colorectal tumor tissue. Immunohistochemical analysis of MLH1 expression was carried out as described before [44].

For SPTAN1 detection, sections (2 μm) of representative samples were cut from paraffin embedded invasive growing colorectal carcinoma specimens. Normal colon mucosa served as an internal control. Sections were deparaffinized three times with Xylene and rehydrated in graded alcohol baths. Antigen retrieval by heating was required in a pressure cooker for 2 min with EDTA buffer, pH 8.0. This was followed by incubation of 5 min with 3% H_2_O_2_ for blocking endogenous peroxidase. Before and between different incubation steps, the sections were washed with 1× Envision Flex Wash Buffer (Dako, Germany). Primary antibody (Santa Cruz; mouse monoclonal antibody clone C-11; dilution 1:250) were diluted in Antibody diluent (Zytomed). Sections were incubated with the primary antibody for 30 min at room temperature followed by application of the EnVision-system (DakoCytomation) with horseradish peroxidase as enzyme and 3,3′diaminobenzidine tetrahydrochloride as chromogen. The sections were counterstained with Mayers hematoxylin (Applichem).

Immunhistochemical staining was examined using a Keyence microscope (Model BZ 9000 for a magnification of 100×, KEYENCE Co., Osaka, Japan).

### Migration assay

Prior to initiating the migration assay, 1×10^4^ cells per well were seeded in 96-well Essen ImageLock plates (Essen Bioscience, Ann Arbor, Michigan, USA) and grown for 48 h to confluence under standard conditions. Then, cell-free zones were generated by using a 96-pin WoundMaker (Essen Bioscience, Ann Arbor, Ml) to simultaneously create wounds in all wells.

Thereafter, the plates were placed inside an automated microscope (IncuCyte™ (Essen Instruments)) which resides inside a standard cell culture incubator and equilibrated for 2 h before the first scan.

The cells were scanned every 3 h and the width of the cell-free zone was determined by IncuCyte™ software which is capable to identify the exact wound region.

To calculate the exact distance of cell migration the detected wound width of each time point was subtracted from the first measured wound width in the corresponding well. This value was divided by 2 and indicates the length (μm) of migration.

In parallel pictures were taken by IncuCyte from each well at every time point. The experiment was performed in triplicate.

## Abbreviations

MMR: DNA mismatch repair; SPTAN1: Non-erythroid spectrin αII; CRC: Colorectal cancer.

## Competing interests

The authors declare that they have no competing interests.

## Authors’ contributions

IH, BPE, FN and SP were involved in performing experiments, acquisition and analysis of data and drafting of manuscript. DS, VS and NF performed acquisition of patients, collection of tissues, analysis and interpretation of data and drafting of manuscript. GP and SZ participated in interpretation of data and helped to draft the manuscript. AB performed conception and design of study, preparation, analysis and interpretation of data, drafting and editing of manuscript. All authors read and approved the final manuscript.

## Supplementary Material

Additional file 1 **Coimmunoprecipitation of endogenous MLH1 and SPTAN1.** Interaction of endogenous MLH1 with SPTAN1 was verified by coimmunoprecipitation (IP). Whole cell extract of MLH1 proficient HEK293 cells was precipitated with protein A/G sepharose and anti-MLH1 or anti-SPTAN1, separated by SDS-page, blotted and proteins were detected. Whole cell extract of HEK293 cells served as expression control (1), precipitation with protein A/G sepharose as negative control (2), IP was performed using anti-MLH1 (3) and anti-SPTAN1 (4). Click here for file

Additional file 2 **Expression increase of MLH1 is reflected by enhanced protein level of SPTAN1.** Direct influence of MLH1 expression on the protein level of SPTAN1 was additionally analyzed using HEK293T cells transiently transfected with MLH1. (A) Whole cell extracts were harvested at different time points after transfection and the expression of MLH1 as well as SPTAN1 was analyzed in parallel by Western blotting using anti-MLH1 or anti-SPTAN1, respectively, controlled by beta-Actin detection. MLH1 was detectable 9 h after transfection (lane 4) and reached a maximum after 20 h (lane 9). (B) MLH1 expression was quantified and correlation of MLH1 level was performed by setting maximum of MLH1 after 20 h as 100%. Slightly time-delayed, an increase of SPTAN1 was visible 14 h after transfection of MLH1 (lane 6). (C) Quantification of SPTAN1 level was performed and assessed by setting SPTAN1 expression, 20 h after transiently transfection of MLH1, as 100%. Graphs indicate the results (mean ± S.D.) of at least three independent experiments. Click here for file

Additional file 3 **SPTAN1 expression is not reduced in MSH2 deficient LoVo cells.** To exclude a potential influence of MSH2 on SPTAN1, the expression of SPTAN1 was determined in MSH2 deficient (mock control transfected) in comparison to MSH2 overexpressing LoVo cells and in HeLa cells. 48 h after transfection cells were harvested and protein extracts were analyzed by Western blotting using anti-MLH1 or anti-SPTAN1, respectively, controlled by beta-Actin detection. As shown by our data, MSH2 has no influence on the expression of SPTAN1. Graphs indicate the results (mean ± S.D.) of at least three independent experiments. Click here for file
